# Painless Destruction of Nævi

**Published:** 1889-08

**Authors:** 


### Painless Destruction of Nævi.

A. B., aged 2. First painted the healthy skin around the circumference of the nævus, for about half an inch, with a coating of collodion flexile; a thick layer of a 4 per cent. solution of corrosive sublimate in collodion was applied over the nævus. The twelfth day, collodion was removed; the nævus had entirely disappeared.

